# Ground by Status

**DOI:** 10.1007/s11098-023-02093-4

**Published:** 2024-01-22

**Authors:** Lisa Vogt

**Affiliations:** 1 Department of Philosophy, University of Geneva, Geneva, Switzerland; 2 Department of Philosophy, Freie Universität Berlin, Berlin, Germany

**Keywords:** Grounding, Logic of grounding, Explanation, Essence, Necessity

## Abstract

What is the explanatory role of ‘status-truths’ such as essence-truths, necessity-truths and law-truths? A plausible principle, suggested by various authors, is Ground by Status, according to which status truths ground their prejacents. For instance, if it is essential to *a* that *p*, then this grounds the fact that *p*. But Ground by Status faces a forceful objection: it is inconsistent with widely accepted principles regarding the logic of grounding (Glazier in Philos Stud 174(11):2871–2889, 2017a, Synthese 174(198):1409–1424, 2017b; Kappes in Synthese 199(1–2):2575–2595, 2020, Philos Stud 178(4):1267–1284, 2021). I defend Ground by Status against this objection.

Consider the following statements:

It is essential to singleton Socrates that it have Socrates as a member.

By metaphysical necessity, there is a first moment in time.

It is a law of metaphysics that any two objects compose another object.

According to these statements, the embedded claims—the prejacents—enjoy a certain ‘robust’, not merely accidental, status: the status of pertaining to the essence of some entity, holding with metaphysical necessity, or being a metaphysical law, respectively.^1^ Let us, borrowing terminology from Kappes (2020), call the truths expressed by true such statements ‘status truths’. Apart from the cases mentioned, the category of status truths also includes truths regarding logical and natural necessity, as well as logical and natural laws.

Status truths are factive: in all cases in which a status truth holds, so does its prejacent. But more than that: many philosophers have been drawn to the idea that status truths do not merely correlate with the truth of their prejacents, but explain them.^2^ And indeed, our explanatory practises seem to accord with this idea. If asked ‘Why is it that singleton Socrates contains Socrates?’, one natural response seems to be: ‘That is just what singleton Socrates *is*–it has Socrates as a member by its very essence’. When confronted with the question ‘Why is there a first moment in time?’ some philosophers may feel tempted to reply ‘Well, it just could not have been otherwise, there simply *has to be* a first moment in time’. And a metaphysician who countenances universal composition as a law of metaphysics may want to answer ‘because it is a law of metaphysics that any objects do so’ when called upon to explain how it comes that her laptop and cat compose another object. Assuming that status truths explain their prejacents, the natural next step is then to understand these explanations in terms of ground. After all, the relevant explanations look distinctively metaphysical in character, and grounding-explanations are commonly taken to be paradigmatic cases of metaphysical explanations. Putting these considerations together, we arrive at the principle Ground by Status. According to this principle, status truths ground their prejacents and if the prejacents are universally quantified (such as in the case of the law of universal composition), also instances of their prejacents.^3^

Surely, Ground by Status is not the only way to go in reaction to the example cases. For one thing, one could postulate additional forms of metaphysical explanation distinct from ground, an option explored by Glazier (2017a), Glazier (2017b) and Kappes (2020), Kappes (2021). And for another, one could contest the view that the example cases correspond to genuine explanations. Going that route, one might e.g. hold that the answers in the cases correspond merely to ways to reject the need for explanation, or that they provide some of what we desire from explanations—such as surprise reduction—while falling short of providing proper explanations.^4^ But while Ground by Status is not the only option, it is the natural default theory of the explanatory role of status truths, and comes with various theoretical benefits. It offers a particularly straightforward, simple and uniform account of the example cases. It can account for the fact that status truths are factive, and even necessarily so, in in a smooth and natural way. For given that grounds necessitate what they ground, the necessitation of prejacents by the status truths immediately follows. And Ground by Status does not demand the introduction of any novel resources, but makes do with the well researched notion of grounding.

Glazier and Kappes have recently made a forceful and highly general case against Ground by Status, however. They think that Ground by Status has to be rejected for all kinds of status-truths: for essence-truths (Glazier, 2017a; Kappes, 2020), necessity-truths (Glazier, 2017b; Kappes, 2020), and law-truths (Kappes, 2020, 2021). One argument that plays a crucial role in both Glazier’s and Kappes’s case against Ground by Status is an objection that I shall label ‘the argument from the logic of ground’, or, for short, ‘the LG-argument’. The gist of this objection is that, granting the existence of plausible candidates for status truths, Ground by Status violates an intuitively plausible and widely held view: that any ground for a disjunction has to be ‘mediated through’ the disjuncts, in a sense to be specified later on.^5^

My aim in this paper is to defend Ground by Status against this argument. I start out by presenting the LG-argument in some more detail (§1). I then show that, on closer examination, the principle about the grounds of disjunctions that the LG-argument rests upon is incompatible with a worldly conception of ground, viz., a conception on which grounding is purely sensitive to how the world is in itself, independent of the way in which we represent it. Hence, for the LG-argument to succeed, a case would need to be made that Ground by Status has to be understood in terms of the alternative representational conception of ground, according to which ground is also sensitive to our representational guises. But there are no good reasons to think that a representational construal of Ground by Status is mandatory. Thus, proponents of Ground by Status should construe it as a principle about worldly grounding, escaping the LG Argument (§2).

## The argument from the logic of ground

To remain neutral on the question of whether grounding is to be understood as a relation between entities, I will take grounding claims to be officially regimented in terms of a sentential operator ‘<’. Here, ‘$A < B$’ may be approximated by formulations such as ‘its being the case that *A* makes it the case that *B*’ or ‘*B* because *A*’ in natural language. Despite officially using the operationalist framework, I shall often nevertheless speak as if grounding was a relation between truths (‘*A* grounds *B*’ and the likes) to facilitate formulations in natural language. I presume a factive understanding of ground, i.e., one on which for ‘$A <B$’ to be true, both ‘*A*’ and ‘*B*’ have to be true. Moreover, I shall use the word ‘ground’ in the sense of ‘strict, full ground’, as opposed to ‘weak/partial ground’.^6^

As anticipated in the introduction, the goal of the LG-argument is to show that Ground by Status contradicts a plausible and influential view on the grounds of disjunctions: the view that, to borrow Kit Fine’s (2012a) phrase, any ground of a disjunction has to be ‘mediated through’ its true disjuncts. The thought is this. When investigating into the grounds of disjunctions, a natural starting point is that disjunctions are grounded in their true disjuncts. For instance, the truth that Barcelona is in Spain or Barcelona is in Antarctica is grounded in the truth that Barcelona is in Spain, and the truth that it is sunny or it is cloudy is grounded in, say, the truth that it is sunny. Yet the demand that disjunctions should be exclusively grounded in their true disjuncts is too strong and needs to be loosened. Thus, to avoid violations of the transitivity of grounding, one should also countenance the grounds of true disjuncts as grounds of disjunctions. And indeed, following Fine (2012ab), one may think that a disjunction can also be grounded in truths which stand neither in a relationship of identity nor of ground to one of the disjuncts, but something which, to put it crudely, also encompasses cases ‘in between’ the two. The idea here is that there might be cases in which one truth is distinct, but so closely connected to another truth that the former truth can do all the grounding-work of the latter truth: whatever the latter truth can ground, the former can too. To give an example, one might think that the truth that the cat is on the mat is so closely related to the truth that the mat is under the cat that the former can ground whatever the latter can ground. Let us say that in such cases, the former truth ‘subsumes the grounding-work’ of the latter truth. That is, *A* subsumes the grounding-work of *B* iff, for any $C_1, C_2,...$ and *D*, if $B, C_1, C_2,....$ ground *D*, then $A, C_1, C_2,...$ ground *D*.^7^ Note that, given this definition, any truth automatically subsumes the grounding-work of itself and given the transitivity of grounding, any truth that grounds another truth also subsumes the grounding-work of this truth. So we can state the condition directly in terms of grounding-work subsumption. Finally, there is a further option that one may additionally want to countenance: that disjunctions can also be grounded in truths that ground the conjunction of their true disjuncts. Adopting all of these ideas, we obtain:

Disjunctions: If *B* grounds $A_1 \vee A_2$ then either (a) $A_1$ is true and *B* subsumes the grounding-work of $A_1$, or (b) $A_2$ is true and *B* subsumes the grounding-work of $A_2$, or (c) $A_1 \& A_2$ is true and *B* grounds $A_1 \& A_2$.^8^

As Glazier notes, Disjunctions follows from the elimination rule for disjunctions in Fine’s (2012a) influential logic for grounding. And indeed, various other logics of grounding that have been suggested later on accord with the principle (see Correia (2017a, 2018, deRosset and Fine (2022), Krämer (2018, 2019)).^9^Prima facie, there are thus strong reasons to adopt Disjunctions: it is suggested by intuitive considerations when trying to precisify the idea that the grounds for disjunctions have to be mediated via their disjuncts, and it is backed up by its incorporation into these broader formal theories.

The goal of the LG-argument is now to show that, in the presence of Disjunctions, Ground by Status conflicts with plausible example cases of status truths with disjunctive prejacents. In the case of essence, the argument can be illustrated on Glazier’s example of a specific Boolean variable foo in a computer program. foo has essentially value 0 or value 1. But it has neither one of these values essentially—if the data input in the program differed, foo could have a different value than it actually has, and foo changes it value over the course of time, or so we may assume. To fix ideas, let us assume that foo actually has value 1. Now, combining Disjunctions and Ground by Essence (the restriction of Ground by Status to the case of essence), a conflict arises in the case of foo. Letting ‘$\Box _{a}$’ stand for ‘it is essential to *a* that’, we have:

(E) $\Box _{\texttt{foo }}$ (foo has value 0 or foo has value 1).

By Ground by Essence, (E) grounds:

(D) foo has value 0 or foo has value 1.

Disjunctions in turn dictates that (E) subsumes the grounding-work of the true disjunct, viz., of:

(D1) foo has value 1.

But this cannot be the case. For (D1) grounds, among others, contingent truths, such as plausibly the following one:

(D1^′^) foo has value 1 or Biden is US president in 2022.

If (E) were to subsume the grounding-work of (D1), it would thus have to ground (D1^′^) as well. Given that grounds necessitate what they ground, however, whatever is grounded in a necessary truth will be itself necessary. And in consequence, necessary truths such as (E) can never ground contingent truths such as (D1^′^).^10^ In other possible worlds in which foo has value 0 and someone else is US president in 2022, (E) still obtains, but (D1^′^) does not—which would, if (E) were to ground (D1^′^), violate grounding necessitarianism.

Abstracting away from the case of foo and essence, the LG-argument can be seen as having the following general form:

1. Disjunctions.
2. There are cases of status-truths of the relevant type with a disjunctive prejacent, such that none of the disjuncts is (metaphysically/naturally) necessarily true.
3. Status truths of the relevant type are (metaphysically/naturally) necessarily true, and grounds (metaphysically/naturally) necessitate what they ground.
  $\therefore$ The relevant type of Ground by Status is false.

Let us make a number of common assumptions: that the laws of logic/metaphysics/nature are logically/metaphysically/naturally necessary, respectively; that essence entails metaphysical necessity; and that logical necessity entails metaphysical necessity, which in turn entails natural necessity. Then, the argument can be run in terms natural necessity in the cases of Ground by Natural Necessity and Ground by Natural Law, and with either natural or metaphysical necessity in all the other cases. And we can use the case of foo not only in an argument against Ground by Essence, but also in an argument against Ground by Metaphysical Necessity and Ground by Natural Necessity. A further example offered by both Glazier and Kappes is the law of excluded middle, according to which, for any *p*, $p \vee \lnot p$. This law serves as an example in the case of Ground by Logical Law, and suitable instances of it serve as examples in the cases of Ground by Logical/Metaphysical/Natural Necessity. However, the cases of Ground by Metaphysical Law and Ground by Natural Law are still remaining. And these cases are trickier—there are no example cases offered in the literature, and I do not have convincing cases to offer either.^11^ So I will have to leave the question of whether the LG-argument applies to these forms of Ground by Status open here.

## Against the argument from the logic of ground

In the remainder of the paper, my aim will be to challenge the LG-argument against Ground by Status. My objection will neither touch upon the relevant example cases, which I find myself plausible, nor on the modal principles employed in the argument, which I am happy to grant. Instead, my only target in what follows will be the principle Disjunctions. For while, at first glance, this principle enjoys a high degree of intuitive appeal and theoretical support, at second glance, there are independently motivated reasons to resist it.

In a nutshell, the argument will be this. On closer examination, the principle Disjunctions turns out to be incompatible with a natural and popular conception of grounding, according to which grounding is a *worldly* phenomenon. Disjunctions is only compatible with the alternative *representational* conception of grounding. But there are no good reasons to think that proponents of Ground by Status should conceive of grounding along representational rather than worldly lines, and thus the LG-argument fails to make a convincing case against Ground by Status.

The argument will proceed in a number of steps. To start, let us assume that Emma, Chris and Mary are cups on my kitchen shelf that are emerald, crimson and maroon all-over, respectively. Now consider the following grounding claim:

(G) Emma is white or Chris is crimson < Emma is white or (Chris is crimson or Mary is white).

This grounding claim is incompatible with Disjunctions. For, according to Disjunctions, (G) could only be true if the putative ground, viz.,

(1) Emma is white or Chris is crimson,

were to subsume the grounding-work of the true disjunct of the groundee, viz.:

(2) Chris is crimson or Mary is white.

And this cannot be the case. For (2) grounds truths that (1) does not ground, such as, plausibly:

(2^′^) (Chris is crimson or Mary is white) or Biden is US-president in 2022.

To see this, we can draw again on the consideration that grounds necessitate what they ground. For, clearly, (1) fails to necessitate (2^′^). In worlds in which, say, Emma and Chris are both white, Mary is maroon and the US-president in 2022 is Sanders, (1) will still be true, but (2’) will not. So (G) is incompatible with Disjunctions.

But now, consider the following plausible grounding claim which is in perfect harmony with Disjunctions:

(G*) Emma is white or Chris is crimson < (Emma is white or Chris is crimson) or Mary is white.

The only difference between (G*) and (G) consists in the way in which the sentence-atoms are arranged in terms of brackets. Starting from the groundee in the case of (G*), we can arrive at the one in (G) simply by moving the brackets. Hence, if (G*) is true but (G) is false, this operation fails to preserve ground-theoretic role. Let us say that *A* and *B* are *ground-theoretically equivalent* if they play the same ground-theoretic role, viz., ground the same truths and are grounded in the same truths.^12^ We can thus see that, in order to endorse Disjunctions, one has to reject the following principle:

G-Associativity: For any *A*, *B* and *C*: $(A \vee B) \vee C$ and $A \vee (B \vee C)$ are ground-theoretically equivalent.

However, there are reasons to think that, for it to be the case that $(A\vee B) \vee C$ *just is* for it to be the case that $A \vee (B \vee C)$. That is, the difference between any sentence of the form $(A\vee B) \vee C$ and the corresponding sentence of the form $A \vee (B \vee C)$ is plausibly a purely representational one. While the two sentences differ with regard to their syntactical form, they still express the same ‘chunk of reality out there’: they represent reality as being the very same way, only under different representational guises. Let us say that *A* and *B* are *worldly equivalent* if for *A* to be the case just is for *B* to be the case in this sense.^13^ Then, the claim can be expressed as follows:

W-Associativity: For any *A*, *B* and *C*: $(A\vee B) \vee C$ and $A \vee (B \vee C)$ are worldly equivalent.

In the recent literature, there has been a surge of interest in the ‘just is’-idiom, and various accounts of it have been proposed (see e.g. Bacon and Dorr (forthcoming), Brast-McKie (2021); Correia (2010), Correia (2016); Dorr (2016); Elgin (forthcoming); Linnebo (2014); Rayo (2013)). These accounts depart from various different theoretical starting points and arrive at substantially different logics governing the idiom. Yet all of these accounts are in agreement that for it to be the case that $(A\vee B) \vee C$ just is for it to be the case that $A \vee (B \vee C)$. In order to reject this principle, one needs to uphold an extremely fine-grained view on reality—which, once properly spelled out, notoriously threatens to lead us into the Russell-Myhill paradox.^14^ While offering a proper assessment of W-Associativity is beyond the scope of this paper, I take there to be strong prima facie reasons in its favor and I shall assume it in what follows.

Provided that W-Associativity holds, however, it directly follows that, in order to reject G-Associativity, one has to maintain that worldly equivalent truths can still come apart with regard to their ground-theoretic roles. That is, one has to reject:

Worldliness of Ground: Worldly equivalence implies ground-theoretical equivalence.

Thus, in the presence of W-Associativity, proponents of Disjunctions cannot uphold a view on which grounding is purely sensitive to what reality is like in itself. Instead, they are forced to endorse a more fine-grained view on which grounding is also sensitive to the particular ways in which we conceptualize reality.

Following the common terminology of the debate on grounding, let us call a conception of grounding that countenances Worldliness of Ground a ‘worldly conception’ of grounding, and a conception that does not a ‘representational’ (or ‘conceptualist’) conception of grounding.^15^ Accounts of grounding that either explicitly state that they concern a worldly conception of grounding or which incorporate grounding-principles that plausibly correspond to a worldly conception include (Audi 2012a, b; Correia (2010, forthcoming); Correia and Skiles (2019); Fine (2012a, 2012b (semantic side), 2017a; and Lovett (2020). Accounts of grounding that correspond to a representational conception include Correia (2017a, 2017b, 2018; deRosset and Fine (2022); Fine (2012a)b (proof-theoretic side); Krämer (2018), 2019; Rosen (2010); and Schnieder (2010).^16^

To be perfectly clear, an account of grounding counts as representational iff it holds that *some* purely representational feature or other is relevant for difference in ground-theoretic status. But this does not mean that proponents of a representational conception need to conceive of *all* representational features as relevant for ground-theoretic status. And thus, although virtually all extant accounts of representational grounding do conceive of the arrangement of brackets as relevant for ground-theoretic status and reject G-Associativity, they need not do so.^17^ My point here is thus merely that, *if* an account of grounding is worldly, it has to endorse G-Associativity on pain of having to give up on W-Associativity, not that only worldly accounts may endorse this principle,^18^

Let me further illustrate the distinction between worldly and representational grounding by some example cases. On a worldly conception of grounding, the following three grounding claims are arguably plausible:

(G2) Emma is emerald or Chris is crimson < Emma is green or Chris is red.

(G3) Emma is emerald or Chris is crimson < Something is emerald or crimson.

(G4) Something in emerald < Something is green.

On representational accounts that incorporate Disjunctions, by contrast, these claims turn out to be problematic.^19^ (G2) proves incompatible with Disjunctions, following a reasoning parallel to the one in the case of (G). And (G3) and (G4) would be ruled out by a principle analogous to Disjunctions for the case of existential generalizations, according to which the only grounds for existential generalizations are (conjunctions of) truths that subsume the grounding-work of instances plus possibly totality truths.^20^

But there are also grounding claims where the situation is reversed: claims that are plausible on a representational conception of grounding but are problematic on a worldly conception. Thus, on a representational conception, it is commonplace to maintain that every true disjunction is grounded in each of its true disjuncts, and every true conjunction in all its conjuncts taken together. And, as limiting cases, this yields:

(G5) Emma is emerald < Emma is emerald and Emma is emerald.

(G6) Emma is emerald < Emma is emerald or Emma is emerald.

On a worldly conception, by contrast, these two claims are arguably to be rejected. For, plausibly, in both claims, the sentences expressing ground and groundee represent reality as being in the same way and merely differ with regard to their representational guises. Hence, on pain of getting violations of the irreflexivity of grounding, the proponent of worldly grounding should reject the idea that every true disjunction is grounded in its true disjuncts and every true conjunction in its conjuncts taken together. Instead, she would uphold restricted versions of these principles that exclusively concern standard cases in which, to put it crudely, the disjuncts/conjuncts are suitably ‘disconnected’.^21^

With these considerations in place, let us now return to the case of Ground by Status. As we have seen, the situation is this. The LG-argument against Ground by Status rests on Disjunctions. In order to endorse Disjunctions, one has to reject G-Associativity. But making plausible assumptions about worldly equivalence, G-Associativity is mandatory on a worldly conception on grounding. Hence, the LG-argument can only be sustained on a representational conception of grounding, but not on a worldly one. And thus, in order for the LG-argument to succeed, it would need to be shown that Ground by Status *has to be* interpreted in terms of representational as opposed to worldly grounding.^22^

But it is hard to see how such an argument might go. Thus, arguably, the only situations where the representational conception of grounding would be mandatory would be ones in which ground and groundee are worldly equivalent, such as, on certain views, cases in which the grounds provide us with a metaphysical analysis/real definition of the groundee (Correia (2017b); Rosen (2015); Skiles (2014)). And the case of Ground by Status is clearly not one of these cases. For, in the case of Ground by Status, ground and groundee are not worldly equivalent: it is not the case that for foo to have essentially value 0 or value 1 just is for it to have value 0 or value 1; or that for there to be necessarily a first point in time just is for there to be a first point in time; or that for it to be law of nature that objects attract each other with this-and-that force just is for objects to attract each other with this-and-that force, and so on. The relevant status truths demand something of reality that goes beyond what their prejacents do, and they are thus not worldly equivalent to them. Proponents of Ground by Status are thus under no pressure to adopt a representational construal of their principle.

And indeed, the construal of Ground by Status in terms of worldly grounding is not merely a legitimate, but a very natural one: as a claim regarding the objective structure of reality in itself, independent of our representational guises. But, as we have seen, as soon as Ground by Status is interpreted in this way, Disjunctions becomes untenable and Ground by Status immune to the LG-argument. And importantly, the rejection of Disjunctions for Ground by Status on the basis of these considerations is not an ad hoc move, with the only purpose of saving Ground by Status. Instead, the move is independently motivated by the natural and popular thought that (at least one type of) grounding corresponds to a purely worldly relationship. All in all, rejecting Disjunctions on the basis of the worldly vs. representational distinction is an independently motivated and plausible way of blocking the LG-argument. Proponents of Ground by Status should construe it as a principle about worldly grounding, escaping the LG-argument.

## Footnotes

1 Here and in what follows, the expressions ‘necessarily’, ‘necessity’ etc. are reserved for the case of metaphysical necessity, unless mentioned otherwise.

2 For the case of necessity, the claim traces back to Leibniz (1714) and has been defended by e.g. Block and Stalnaker (1999); Biggs (2011); Glazier (2017b); Hill and Mclaughlin (1999); Rundle (2004); and Inwagen and Lowe (1996). For the case of essence, its advocates include Dasgupta (2014); Glazier (2017a); Kment (2014); Lange (2009); Rosen (2010), and for the case of law-truths, Kment (2014); and Lange (2009). This list is mainly drawn from Kappes (2020).

3 We may distinguish the wider version of Ground by Status—that I assume here—from a more narrow principle which merely maintains that status truths ground their prejacents, but not that they ground instances of them. Note also that in the case of essence, Ground by Status is commonly restricted to a ‘narrow’, i.e. immediate constitutive notion of essence.

4 I would like to thank an anonymous reviewer for this suggestion.

5 Note that the LG-argument is not the only argument that has been raised against Ground by Status In particular, Glazier (2017a) also develops a parallel argument for the case of existential generalizations. For some further arguments, see Glazier (2017a); Kappes (2020), Kappes (2021); Van Cleve (2018); and Zylstra (2019). For a response to Zylstra, see Vogt (forthcoming).

6 See e.g. Fine (2012a) on the relevant distinctions.

7 Strictly speaking, we should use higher-order resources to express grounding-work subsumption. Thus, using ‘$\triangleleft$’ as a sentential operator for grounding-work subsumption, and ‘$\forall$’ to express universal quantification into sentence position, we would have: $A \triangleleft B$ iff $\forall C_1, C_2,..., D ((B, C_1, C_2,...< D) \rightarrow (A, C_1, C_2,... < D))$.

8 Glazier can be plausibly interpreted as adopting this principle. Kappes adopts the stricter original principle which does not allow for grounds of the conjunction as grounds, nor for cases of grounding-work subsumption distinct from identity or ground. This principle directly entails Disjunctions.

9 Ignoring complications due to factive vs. non-factive notions of grounding: some but not all of these systems allow for (c) as an option. In all these systems, the relevant rules are stated in terms of the notion of a *weak grounding.* While the notion of weak grounding is understood in somewhat different ways in the systems, in all of them, weak grounding implies grounding-work subsumption. Thus, in all of the systems, the following conditional holds:

- (C) If *A* grounds *B*, then (a) *A* weakly grounds *B* and (b) there are no $C_1, C_2,...$ such that $B, C_1, C_2,...$ ground *A*.

And it can be shown that, by the Reflexivity- and the Cut-principle for weak ground (which hold in these systems), (C) implies that, if *A* weakly grounds *B*, *A* subsumes the grounding-work of *B*.

10 The view that grounds necessitate what they ground and that, in particular, necessary truths cannot ground contingent truths is widely accepted in the debate on grounding. For a critique of grounding-necessitarianism, see Baron-Schmitt (2021), Leuenberger (2014) and Skiles (2015) and for an argument that necessary truths can ground contingent truths, see Amijee (2020). Note, however, that even in the absence of grounding-necessitarianism, it would be highly implausible to claim that the essence-truth should ground (D1^′^). For foes of grounding-necessitarianism can still agree that grounds necessitate what they ground *given that certain background conditions* hold. For instance, foes of grounding-necessitarianism may want to hold that a given universal generalization is fully grounded in all of its instances and that, while these instances do not necessitate the universal generalization, they still necessitate it *relative to the background condition that they are all the instances*. And there simply is no plausible background condition relative to which the essence-truth would necessitate (D1^′^). So while incorporating the assumption of grounding-necessitarianism allows for a more smooth and clear-cut argument, the success of the argument does not ultimately hinge on it. The same will hold in all the other cases where grounding-necessitarianism will be employed in what follows.

11 Here is a more abstract consideration that might tell in favor of the existence of relevant laws of nature, however: One may think that some, or even all prejacents of laws of nature are of the form of a universally quantified material conditional, where the antecedent ‘picks out’ the relevant systems/entities and the consequent then ‘says’ what holds for these systems/entities (cf. Friend (2016)). Assuming that, within the scope of the ‘it is a law of nature that’-operator, material conditionals can be replaced with the disjunction of the negated antecedent and the consequent, this would yield laws of nature of the right form. Note also that the ‘substitutability of conditionals’-assumption might give rise to further examples of disjunctive essence-truths, namely if (some or all) prejacents of essence-truths were to make claims conditional on the existence of the relevant entity, such as: it is essential to Socrates that he be human *if he exists.*

12 More precisely, using ‘$\sim$’ as a symbol for ground-theoretic equivalence: $A \sim B$ iff: (a) $\forall C_1, C_2,... (C_1, C_2,...< A \leftrightarrow C_1,C_2,... < B)$, and, (b) $\forall C_1,C_2,...,D (A, C_1, C_2,...< D \leftrightarrow B, C_1, C_2,... < D)$.

13 I use the relational predicate ‘is worldly equivalent’ to facilitate expression in natural language. Officially, worldly equivalence should be expressed in terms of a sentential operator.

14 An important way to reject W-Associativity that has been suggested by an anonymous reviewer to me is to maintain that the representational difference between $A \vee (B \vee C)$ vs. $(A \vee B) \vee C$ tracks a worldly difference in how the two are ’built’ from *A*, *B*, and *C*. This idea is naturally further cashed out along the lines of a structured account of facts according to which *A* and *B* are worldly equivalent iff they are build up in the same way from the same higher-order constituents. But (similar to corresponding first-order accounts of facts) this account is threatened by a higher-order version of the Russell-Myhill paradox (see Russell (1903) and Myhill (1958) for the original paradox, and Goodman (2017) for a higher-order version). While there are potential ways around the paradox, they all come at high prices. Thus, the standard way around the paradox is to adopt Russell’s (1908) ramified theory of types (cf. Hodes (2015)). But the ramified account is commonly acknowledged to be extremely complex and quite ad hoc. Two recently proposed alternative accounts, due to Fritz (2019) and Whittle (2022), avoid the complexity, yet have i.a. the disadvantage that they do not allow for the existence of a single unified grounding-relation, but, rather, merely a separate relation for every ‘rank’ of truths.

15 See e.g. Correia (2010, 2020, Fine 2017) and Krämer and Roski (2015) on the distinction, and deRosset (2023) for a critique of the distinction. One might initially think that the distinction could be also drawn in terms of the relata of grounding, along the following lines: a conception of grounding is worldly iff it takes the relata of grounding to be worldly entities, such as states of affairs/chunky facts in the sense of deRosset (2023), and a conception is representational iff it takes the relata of grounding to be representational entities, such as propositions/thin facts in the sense of deRosset (2023). But this way of construing the distinction is merely a rough approximation of the worldly vs. representational distinction in terms of ground-theoretic equivalence as assumed here. First, the distinction between the worldly and the representational conception also arises on an ontologically non-committal understanding of grounding that does not countenance relata of grounding. Secondly, even if relata of grounding are countenanced, it is not guaranteed that the ontological distinction lines up with the one in terms of ground-theoretic equivalence. For instance, it would be an in principle tenable view to take the relata of grounding to be propositions and yet to take propositions that correspond to the same state(s) of affairs to play the same ground-theoretic role (cf. Correia (2020)).

16 In the case of Fine (2012a), the proof-theory corresponds to a representational conception, while the semantics corresponds to a worldly conception. In the case of Fine (2012b), the semantics corresponds to a worldly conception, while the proof-theory is neutral on the distinction (since it does not cover the truth-functional connectives).

17 The only potential exception that I know of is the representationalist account sketched in Correia (2017b), which, under plausible assumptions, yields G-Associativity. This is so since, on Correia’s account, representational differences only matter insofar as one representation is more joint-carving than the other. And, arguably, different positions of brackets in disjunctions do not give rise to differences in joint-carvingness.

18 All the aforementioned logics and semantics for worldly ground vindicate G-Associativity or vindicate it once extended in the natural way so as to capture strict grounding (as opposed to merely weak grounding) and the truth-functional connectives.

19 Proponents of representational grounding could resort to an error-theory to explain why (G2)-(G4) might have a ring of truth to them despite being false on their view. Thus, (on both the worldly and the representationalist account) the left-hand sides and the right-hand-sides of the claims have common grounds. The representationalist could now claim that this situation of common ground is easily confused with a situation in which a direct grounding-relationship obtains. The situation would be similar to the one in the causal case, where we are also often prone to confuse relationships of common cause with direct causal relationships. I would like to thank an anonymous reviewer for this suggestion.

20 As mentioned before, Glazier (2017a) develops an objection parallel to the LG-argument for the case of existential generalizations. His argument is based on precisely this principle and is thus equally subject to the objection that I raise here.

21 See e.g. Correia (2010) and Lovett (2020) for proposals of how to regiment the relevant disconnectedness.

22 Alternatively, one could of course try to argue that Ground by Status conflicts with the logic of worldly grounding in different ways. In particular, one could try to modify Disjunctions in such a way that it becomes compatible with the worldly conception, while still ruling out Ground by Status and remaining independently plausible. Or one could try to mount an argument against Ground by Status based on semantic, rather than syntactic considerations. I leave it to foes of Ground by Status to propose such new arguments.
